# Adaptive Renewal of Mountainous Historical Towns Based on the Stability of the Social Network Structure: A Case Study in Chongqing, China

**DOI:** 10.3389/fpubh.2022.867407

**Published:** 2022-04-01

**Authors:** Yaling Shi, Yong Huang, Ran Zhang, Di Jiang, Junxue Zhang

**Affiliations:** ^1^Tourism and Urban-Rural Planning College, Chengdu University of Technology, Chengdu, China; ^2^School of Architecture and Urban Planning, Chongqing University, Chongqing, China; ^3^Key Laboratory of New Technology for Construction of Cities in Mountain Area, Chongqing University, Chongqing, China; ^4^Chengdu Institute of Planning and Design, Chengdu, China; ^5^Bureau of Planning and Natural Resources of Chengdu Municipal Chenghua District, Chengdu, China; ^6^School of Civil Engineering and Architecture, Jiangsu University of Science and Technology, Zhenjiang, China

**Keywords:** social network analysis (SNA), built environment, social ties, *k-core*, historical towns, core conservation areas (CCAs)

## Abstract

The stability of social network structure (SSNS) in historical towns is influenced by changes in built environments and demographic factors. The historical towns in China have evolved into massive rural-urban migration under the rapid urbanization over the past forty years. In this context, many of these historical towns experienced “declining built environment and disintegrating social networks,” which does not contribute to the adaptive renewal of the built environment and social networks in historical towns, as well as the psychological health of residents. This article intends to explore the adaptive renewal of the built environment and social networks of historical towns based on the SSNS. Data on “households” and “social ties” (i.e., kinship, geographic, and job relationship) among households were collected *via* a field survey in seven historical towns in Chongqing, China. K-core models of social network analysis (SNA) were calculated to analyze SSNS. The result shows that the social networks of historical towns with centripetal-shaped structures were more stable than historical towns with divergent-shaped structures. Moreover, spatial layout forms and functions of households might affect the stability of social networks in historical towns. Based on the results of the analysis of SSNS, strategies for adaptive renewal of the built environments and social networks were put forward in two aspects. The built environment, such as the classification of public spaces and service facilities, can be designed based on the k-core indicator for increasing the spatial connection of households in the historical towns. In addition, increased social activities in historical towns with weak SSNS may promote social connection of households, and are also helpful in boosting public health in psychological aspects.

## Introduction

The adaptive renewal of historical towns is a hot topic in urban renewal (1) and planning policy research (2, 3). It can improve property value and economic development (4, 5), as well as strengthen neighborhood and social ties (6, 7). Since 2002, the “Law on the Conservation of Cultural Relics” (revised edition) of China has been stipulating the concept of historical town. It includes precious cultural relics, historical value, or commemorative significance, but completely reflects some traditional features of certain historical periods and local ethnic characteristics as well (8). Historical town was delineated as the core conservation, construction control, and environmental coordination areas to undertake conservation and management. In core conservation areas (CCAs), buildings should be conserved by classification as historical and cultural elements, and new constructions or expansion activities are forbidden. In subsequent years, a series of regulations were promulgated for conservation of historical towns in China, such as “regulations on the conservation of historical cities, towns and villages” (2008) and “the approval method for the preparation and approval of the conservation plan for historical cities, towns, villages and blocks” (2015). They emphasized that natural landscapes, cultural environments, and livelihood environments should be considered in the conservation of historical towns, which is similar to an Italian slogan of “conserve people and houses together” in 1970 (9).

Built environments and social networks of historical towns are suffering for problems because of rapid urbanization of China, such as decay of built environments (10) and fragmentation of spatial patterns, in the process of population flow and commercialization (11). For example, some dilapidated CCAs of historical towns (e.g., Yunshan Town and Nuodeng Town) had only left one-third of the registered population (12). Traditional styles were also replaced by commercial styles of CCAs of historical towns, such as Shuhe and Shuangjiang (13). On the other hand, some historical towns experienced disintegration of kinship or indifference in neighborhood relationship with migration of residents, affecting the sustainable renewal of built environments (14). For instance, from 1987 to 1999, 32.73% of original residents moved out, and 4,051 new ones moved to Lijiang town. The change of resident structure led to kinship among original residents being replaced by the job relationship among new households. Interactions were reduced between the new population and the original residents (15).

In response to these problems, the existing research on China is concentrated on spatial and social aspects of conservation of historical towns (16). At the spatial aspect, studies mainly focused on the texture continuity, style coordination, and building renovation of historical towns, such as the analysis of the thermal environment (17, 18), the conditions of the built environment of the old historic district (19, 20), and the conservation of urban morphology during urban renewal (11). In the social aspect, research has discussed family or community structure (21, 22), relationships among stakeholders, such as residents, governments, developers, and planners (23), population displacement (24), and gentrification of historical towns (25). Accordingly, tools for conservation assessment of historical towns involve geographic information system (GIS), space syntax (SS), and social networks analysis (SNA) (26). The decision model of GIS and the axis model of space syntax emphasized the material spaces of historic towns. Social network models can be built by SNA to visualize the topology of social ties (27).

SNA has been applied to scrutinize changes and characteristics of social ties in rural areas, because social ties play a key role in the mobility of residents (28). It has also been applied to analyze the social ties of settlements from different indicators and applications (29) in traditional settlement (30, 31) and historic conservation areas (29). Social ties covered kinship, neighborhood, and job relationships (32), family generations (33), friendly or antagonistic relationships (34), and complex stakeholder relationships in rural settlement consolidation (35). Specific indicators included network density, lambda set, and degree centrality, characterizing the whole network, clustering feature, and individual characteristics, respectively (36). The *k-core* model of SNA is a commonly used model to measure the stability or engagement of a network (37). SNA is also applied on the guidance of reconstruction of rural settlements (38) and space planning for cultural tourism (39). Consequently, this article would analyze the SSNS of historical towns with the *k-core* model of SNA.

Chongqing is a national historical city in the mountainous areas of southwest China with rich historical and cultural heritage. In Chongqing, a “three-level” conservation system was established for historical and cultural resources (29, 40). However, contradiction between conservation and development of historical towns in Chongqing has been prominent in the context of accelerated urbanization process in the past decades. First, it showed a fundamental tendency of commercialization, resulting in constructive destruction, functional replacement, and disintegration of social ties in historical towns. For instance, commercial trends led to all aborigines of Hongyadong Block of Chongqing moving out. Second, historical and cultural characteristics have heavily disappeared after severe population loss in historical towns. Traditional spatial forms and living environments have also been reshaped along with hollowing. For instance, most aboriginals of CCAs in Ningchang town moved out to seek employment, education, or public services because of the decline in salt industries. Consequently, most permanent residents are aged either <20 years or over 65 years.

Previous studies have indicated that both of built environments and social networks are important in conservation and adaptive renewal of historical towns (8). However, in view of these practical issues, existing studies have focused on the perspective of built environments and neglected social networks in historical towns. Most of them consider that the social networks of historical towns tend to ignore some characteristics of social network structure and its influencing factors. For instance, connection among spatial layout, household functions, and SSNS of historical towns has not yet been involved. They did not also make planning recommendations based on stability of social networks. However, SSNS is related to connectivity and resilience of settlements (36), and is not only an important part of the conservation of historical towns, but also a factor that is always neglected. The SNA approach can be used to visualize the social network structure of residents, and the indicator k-core of SNA can be applied to analyze the SSNS.

Therefore, this article aims to analyze the influencing factor of SSNS in historical towns of Chongqing, and guide the adaptive renewal of built environments and social networks for other historical towns in China. First, according to evaluation results of value characteristics and conservation measures, seven historical towns in Chongqing were selected as samples. Second, data on households and social ties were collected by informant interviews and semi-structured interviews with residents of the studied samples. Third, social network models of the studied samples were constructed with the SNA approach to analyze the characteristics of the social network structure. Fourth, *k-core* indicators of the studied samples were calculated to analyze the SSNS, and influencing factors of the SSNS were discussed. Lastly, strategies for optimizing SSNS in historical towns were submitted.

## Methodologies

### Study Samples

**Figure 1:**
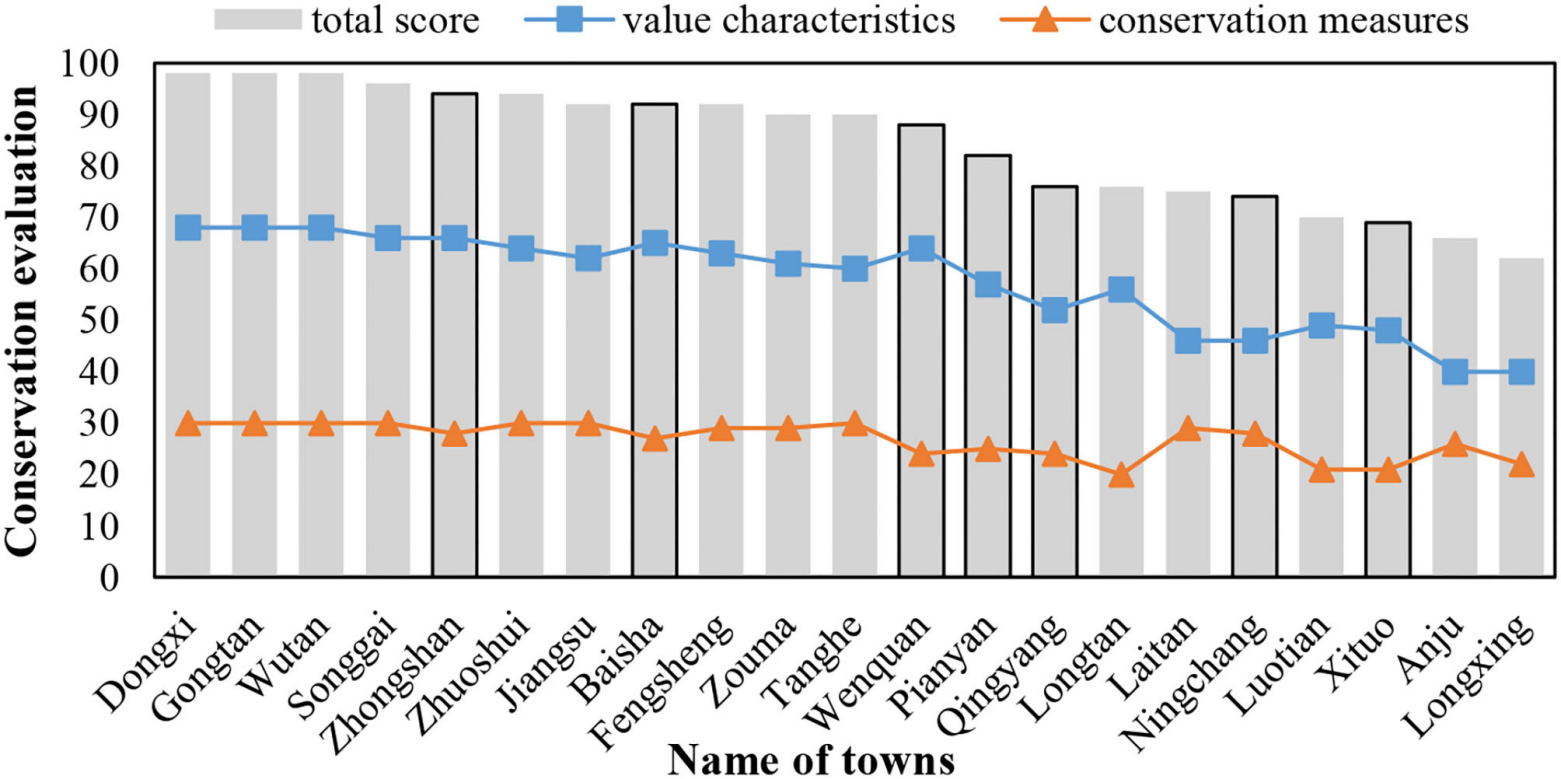
Conservation evaluation of historical towns in Chongqing, China (*n* = 21).

Chongqing has 23 national historical towns, accouting for 12.7% of that in China. The studied samples, the 23 national historical towns in Chongqing, were selected based on quantitative evaluation. The assessment questionnaire was mainly composed of value characteristics of historical resources and conservation measures of historical towns, according to the “evaluation index system of historical towns and villages in China.” In the evaluation questionnaire, value characteristics of historical resources (maximum 70 points) included nine dimensions, namely, D1 (the level and quantity of cultural relics in the built-up area of the town), D2 (the number of historical buildings in the built-up area of the town), D3 (important function characteristics of the town), D4 (size of cultural relics conservation units and historical buildings in the built-up area of the town), D5 (historical environment factors), D6 [size of historical lanes (rivers)], D7 (style integrity, historical authenticity, features of the spatial layout of core conservation areas), D8 (life continuity in core conservation areas), and D9 (intangible cultural heritage). On the other hand, the conservation measures of historical towns (maximum 30 points) included three dimensions, and they are D10 (conservation planning), D11 (conservation and restoration measures), and D12 (guarantee mechanism). The study received 21 valid evaluation questionnaires without Lukong and Shuangjiang towns as they did not take part in the questionnaire assessment. The score results of the conservation evaluation of 21 historical towns in Chongqing are revealed in Figure 1. It can be seen that the total score of 21 historical towns was distributed from 62 to 98 points. By dividing the total score of these evaluations into four grades: 60–70, 70–80, 80–90, and 90–100, one or two towns were selected as study samples in each grade. Based on the criteria, seven historical towns were carefully selected, namely, Zhongshan (ZS), Baisha (BS), Wenquan (WQ), Pianyan (PT), Qingyang (QY), Ningchang (NC), and Xituo (XT).

**Figure 2:**
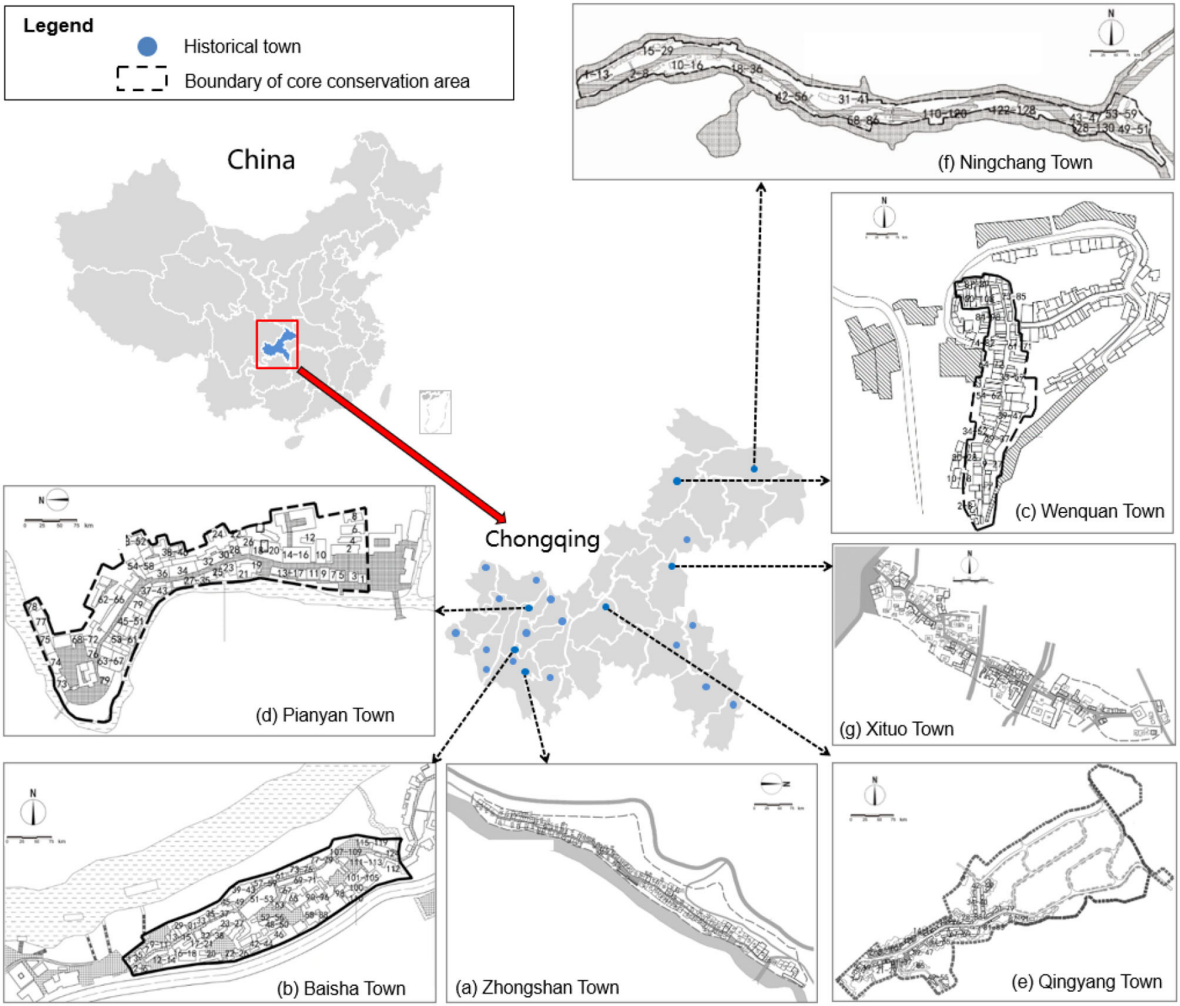
Distribution of 23 national historical towns in Chongqing, China; and layout of the seven studied samples: **(a)** Zhongshan, ZS; **(b)** Baisha, BS; **(c)** Wenquan, WQ; **(d)** Pianyan, PY; **(e)** Qingyang, QY; **(f)** Ningchang, NC; **(g)** Xituo, XT.

Figure 2 presents the distribution of 23 historical towns of Chongqing and layouts of the seven studied samples. CCAs in the seven historical towns are identified as the study area. It can be seen that the layouts of the seven towns are banded owing to being limited by the surrounding mountains and rivers, as shown in Table 1. The layouts of CCAs of ZS, NC, and XT towns are long-banded along the river. The PY town is divided into two groups by the river in the middle of the town, and the layout, as well as the layout of QY town, has a compact-banded shape. The layouts of BS and WQ towns have a group-banded shape. Moreover, the socio-demographics (i.e., CCA, number of households, percentage of four functions of household, and total household) are displayed in Table 1. Number of households was the primary basis for the establishment of social network models. Number of different functions of households was used to analyze the influence factor for SSNS. The functions of households in CCAs of the seven towns were resident-dominated (residents of households > 80%) and resident-business mix types. BS town was dominated by residential function (residents of households >90%). The functions of households included four categories, “resident,” “resident + retail,” “resident + catering,” and “resident+ entertainment” simultaneously in towns ZS, WQ, PY and XT. The households in towns QY and NC had three categories, “resident,” “resident + retail,” and “resident + catering.”

**Table 1:** Brief profiles of the seven studied samples, namely, Zhongshan, Baisha, Wenquan, Pianyan, Qingyang, Ningchang, and Xituo towns.

**Name of town**	**Spatial layout**	**Area of core conservation area (ha)**	**No. of total households**	**Percentages between four types of household's function and total households**			
				**Resident**	**Resident +retail**	**Resident + catering**	**Resident+ entertainment**
ZS	Long-banded	8.1	123	64.23%	16.26%	17.07%	2.44%
BS	Group-banded	14.0	116	98.28%	1.72%	None	None
WQ	Group-banded	12.1	99	68.69%	14.14%	13.13%	2.02%
PY	Compact-banded	4.0	79	59.49%	22.78%	13.92%	3.80%
QY	Compact-banded	12.2	70	67.14%	18.57%	14.29%	None
NC	Long-banded	12.6	98	81.63%	11.22%	7.14%	None
XT	Long-banded	7.8	116	75.00%	12.07%	6.90%	6.03%

### Social Network Analysis

The SNA of the seven studied samples was divided into three steps, data collection of “node” and “line,” construction of social network model, and analysis on SSNS. In the first step, the data of “node” and “line” of the seven samples were collected based on the following semantics explanation. “Node” means one household in the CCA of the historical town. “Line” means social ties among households in the CCA of the town, which consists of three categories in terms of kinship, geographic, or job relationship. In the second step, the collected data on households and social ties in the seven studied samples were converted into an adjacency matrix consisting of “0” and “1.” Among them, “0” means that no social ties among households exist, while “1” indicates that a social tie among the households may exist. The topologies of social networks for the seven studied samples were generated by SNA software (UCINET, version 6.0.). Lastly, the indicator of *k-core* (*k*=*1, 2, 3..*.) was used to calculate the SSNS in seven studied samples. The *k-core* is a condensed subgroup analysis on a social network, and *k* indicates the number of connections of each node in that subgroup to the other nodes (41). The higher the *k*-value and percentages of the *k-core*, the more stable the social network structure. Based on established social network models, the values and percentages of *k-core* were calculated by the *k-core* distribution topology and partitioning map, which was generated by the analysis of “network” → “regions” → “k-core” of the UCINET software. In a *k-core* partitioning map, horizontally represents the node of household while vertically represents the degree of social ties between households.

### Data Collection

Collection of node data for households and line-data for social ties among households in the seven studied samples is needed for the construction of a social network model. First, the number of households was recorded by the field survey, and location information of households was noted on the corresponding layouts as serial numbers. In total, 123, 116, 79, 50, 70, 99, 98, and 116 nodes data of households in the seven studied samples, ZS, BS, WQ, PY, QY, NC, and XT, were collected. Furthermore, line-data for social ties of kinship, geographic, and job relationship among households were collected by informant interviews and semi-structured interviews with the residents. The informant interviews were used to understand the integral information of social ties among households in the CCA of the historical town. The informants in this study were people who have a good understanding of the historical origin, development process, and current situation of each town, including town government staff, head of neighborhood committees, and the elderly with prestige (42). Each town identified 2 or 3 informants. In addition, the semi-structured interviews with residents in each studied sample were utilized to obtain more information. The interviews included two main questions: Q1 (How long have you lived in the CCA of this historical town (residential time)?' and Q2 (Which neighbors (households) in the CCA of this historical town have a social tie (kinship, geographic, or job relationship) with you for more than 10 years?). The recorded relationships were collected as line-data for social ties among households. In the seven studied samples, 1,764, 1,080, 886, 318, 512, 914, 592, and 854 line data were collected, respectively.

## Results

### Analysis of the Stability of Social Network Structure

**Figure 3:**
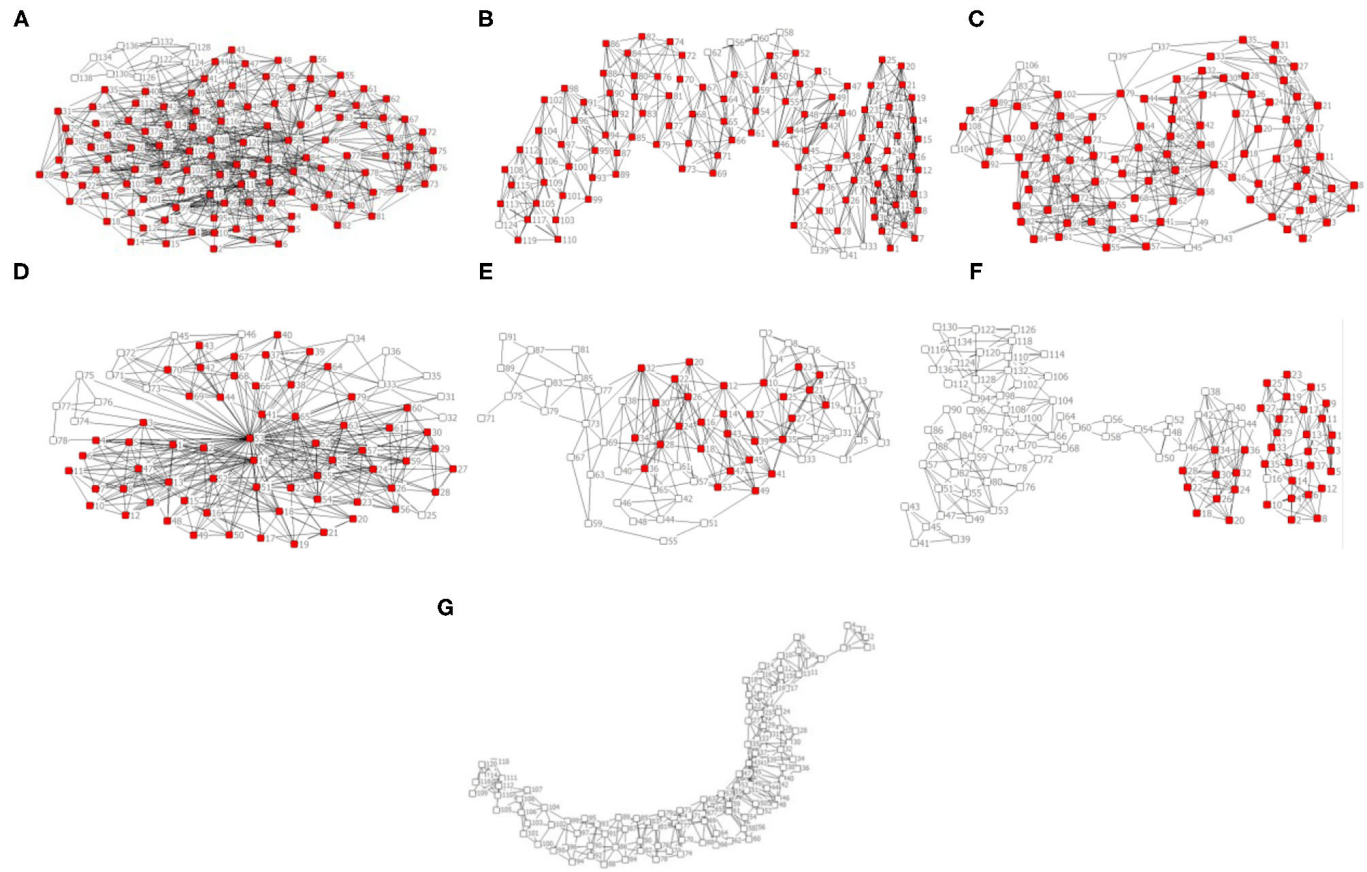
Distribution topology of 6-core of historical towns (red color≥ 6-core, white color <6-core). **(A)** Zhongshan, ZS; **(B)** Baisha, BS; **(C)** Wenquan, WQ; **(D)** Pianyan, PY; **(E)** Qingyang, QY; **(F)** Ningchang, NC; **(G)** Xituo, XT.

The *k-core* indicator was used to analyze the SSNS. Table 2 demonstrates the results of the *k-core* analysis of seven studied towns, including the number of k-core partition, number of households in each k-core partition, and percentages of no <6 core partitions. It can be seen that the maximum value of *k-core* was 10 in towns ZS, BS, or PY, in which the percentage of 10-core of ZS town approached to 70%, whereas the maximum value of *k-core* was only 5 in XT town; it means that the number of social ties among households was limited. Therefore, *6-core* household was regarded as the comparison point of SSNS in the seven studied samples. The SNSS for seven historical towns was compared based on the sorting order of percentages of no <*6-core*, namely BS town (93.04%) > ZS town (92.68%) > PY town (78.48%) > WQ town (89.9%) > QY town (41.43%) > NC town (36.73%) > XT town (none). The results show that the SSNS of households in BS town was highest while that of XT town had 6-core component and that it has lowest stability. Furthermore, the distribution topologies of *k-core* analysis are shown in Figure 3, in which the red color points to households with no <6-core while the white-color points to those with <6-core. It also illustrates that the social networks of historical towns with centripetal-shaped structures (i.e., ZS, BS, WQ, PY, and QY towns) were more stable than historical towns with divergent-shaped structures (i.e., NC and XT towns).

**Table 2:** Results of the *k-core* analysis of seven historical towns.

**Name of town**	**No. of *k-core* partition**	**No. of households in each *k-core* partition**	**Percentages of no <*6-core***	**Shape of social network structure (feature)**
ZS	8	3-core: 1, 4-core: 3, 5-core: 5, 6-core: 1, 7-core: 5, 8-core: 17, 9-core: 9, 10-core: 83	92.68%	Ring (centrality)
BS	5	4-core: 1, 5-core: 7, 6-core: 79, 7-core: 18, 10-core: 13	93.04%	Multi-cluster (centrality)
WQ	6	2-core: 1, 3-core: 1, 4-core: 3, 5-core: 4, 6–core: 21, 7-core: 68	89.90%	Multi-cluster (centrality)
PY	9	2-core: 2, 3-core: 7, 4-core: 2, 5-core: 6, 6-core: 15, 7-core: 7, 8-core: 19, 9-core: 9, 10-core: 12	78.48%	Ring (centrality)
QY	6	1-core: 1, 2-core: 5, 3-core: 14, 4-core: 8, 5-core: 13, 6-core: 29	41.43%	Ring (centrality)
NC	5	2-core: 2, 3-core: 49;4-core: 1;5-core: 10;6-core: 36	36.73%	Series-dendritic (divergence)
XT	3	3-core: 2, 4-core: 44, 5-core: 70	0.00%	Fan-dendritic (divergence)

*“No. of k-core partition” represents the number of cohesive subgroups formed in the social network structure of historical towns*.

### Influencing Factors for the Stability of the Social Network Structure in Historical Towns

#### Spatial Layout Forms

The comparison between layout (Figure 2) and social network structure (Figure 3) of the seven historical towns revealed that the SSNS of the historical towns could be influenced by spatial layout form. As the spatial layout form of the historical towns might be limited by the terrain of mountains and rivers, which has a great impact on the construction of social ties among households, thus affecting the SSNS. In general, the SSNS of historical towns with a group-banded layout was greater than that with a compact-banded or a long-banded layout. It might be that the social ties of households in historical towns with a group-banded layout affected by the terrains were less than those of households in towns with a more compact layout, whereas, households that lived in historical towns with a compact-banded or a long-banded layout were more easily affected by the mountains and rivers and can hardly build social connections with others. But ZS town, which had a long-banded layout, had the second highest SSNS in the seven studied samples, which might be the other factor that holds the social ties of households stable.

BS town or WQ Town, which had a group-banded layout, reflected higher SSNS and presented a multi-cluster shape with centrality (Figures 3B,C). It indicates that the social ties of most households in towns with a group-banded layout were dominated by the geographic and neighbor relationships in the same group. The fellow-townsman relationships of cross-geographical were also partially included when the terrain impact was negligible, such as the relationship between eastern household WQ78 and the western households WQ98 or WQ100 in the WQ Town.

The SSNS of PY town (Figure 3D) and QY town (Figure 3E) with compact-banded layout was moderate. Social networks of the two towns revealed a ring shape with centrality, in which one key-core or multi-key-core households as well as some dispersed households in the town existed. They collectively influence the SSNS of the historical towns. As shown in Figure 3D, the household PY53 was a key-core of the centripetal-shaped social network in the PY Town with a compact-banded layout. It is located in the middle of the town, which enables more social connections with another household around and even in the whole CCA. The households residing on the edge of PY town, such as the north-western household PY78, are relatively unfamiliar with other households in the CCA.

Apart from ZS town (Figure 3A), towns with a long-banded layout exhibited lower SSNS with divergent shapes. Social network structures of NC town (Figure 3G) and XT town (Figure 3H) has a series-dendritic shape and a fan-dendritic shape, respectively. Social ties of the two towns were dominated by neighbor relationships rather than fellow-townsman or job relationships because of the division of terrain or rivers. In NC town, the social tie was allocated to the households on the south side of the CCA, while it was weak among the households in the middle of the town. In XT town, the division of terrain also obstructed the daily communication among households in the town, thus affecting the construction of social connection and SSNS. On the other hand, the layout of ZS town was long-banded, but the structure of social network was ring-shaped centrality. It has multi-core households, and only some households located on the edge have weak geo-relationships with other households, such as ZS138 and ZS134 in the corner of the southwest.

#### Function of Households

Function of household was another factor that affects the SSNS in historical towns on the contrastive analysis of the function of household (Table 1) and SSNS (Table 2). Construction of social ties was influenced by the residential and commercial functions of household, thus affecting the SSNS.

BS town, where residential function dominated 93.04%, had the highest SSNS (percentages of no <*6-core* was 98.28%). As original households of all ages still live in the CCA of BS town, especially most middle-aged and elderly households who have been close neighbors or fellow townsmen for decades. It can be seen that employment opportunities, convenient transportation, and complete infrastructure can be provided for households living in the CCA of the town.

Towns ZS, WQ, PY, or XT has formed mixed functions of “resident,” “resident + retail,” “resident + catering,” and “resident+ entertainment” because of developing handicraft and tourism in the CCA of each town. The employment demand of local residents could be satisfied by the viable tourism. Employed local residents could also retain the relatively stable social ties and SSNS of the historical towns. Moreover, due to the migration of the households of PY Town from the CCA to the new town across the river, the SSNS of CCA of PY was less than ZS Town and WQ Town. Though XT Town is also a highly commercialized town under the developer-led development. The SSNS of XT town was the least in the seven studied samples. A potential reason could be the excessive commercialization caused frequent population migration and instability of social ties among households.

The function of household in CCAs of QY town and NC town consisted of three categories, namely, “resident,” “resident + retail” and “resident + catering.” Because the remaining households was mostly middle-aged and elderly, social activities in QY Town were occasional, and the SSNS of QY town was loose. Similarly, because of remote location, inconvenient transportation, and incomplete public facilities, many young and middle-aged household members have moved out of NC town, leaving the elderly to maintain the living function of the town. Nonetheless, social ties among households were estranged gradually as a result of minor public activities in the CCA and the long distance among the remaining households in the town.

## Conservation Strategy of Social Network Structure in the Historical Towns

**Figure 4:**
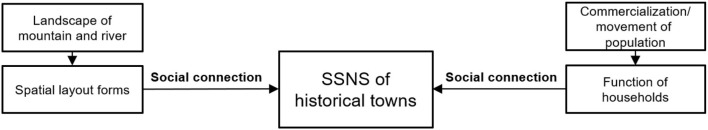
Influencing factors for the SSNS of historical towns.

According to the results, the spatial layout form and function of household are two main factors affecting the SSNS. Because of weaker spatial connection, the SSNS of historical towns with an extensive and compact-banded shape was lower. In addition, the social connection among households would be reduced by the replaced functions of commercialization in historical towns. To improve the conservation of SSNS in the historical towns, the social ties among households need to be increased by enhanced spatial connection and social activities in the built environment and social networks, as shown in Figure 4.

### Spatial Connection Planning in the Built Environment

**Figure 5:**
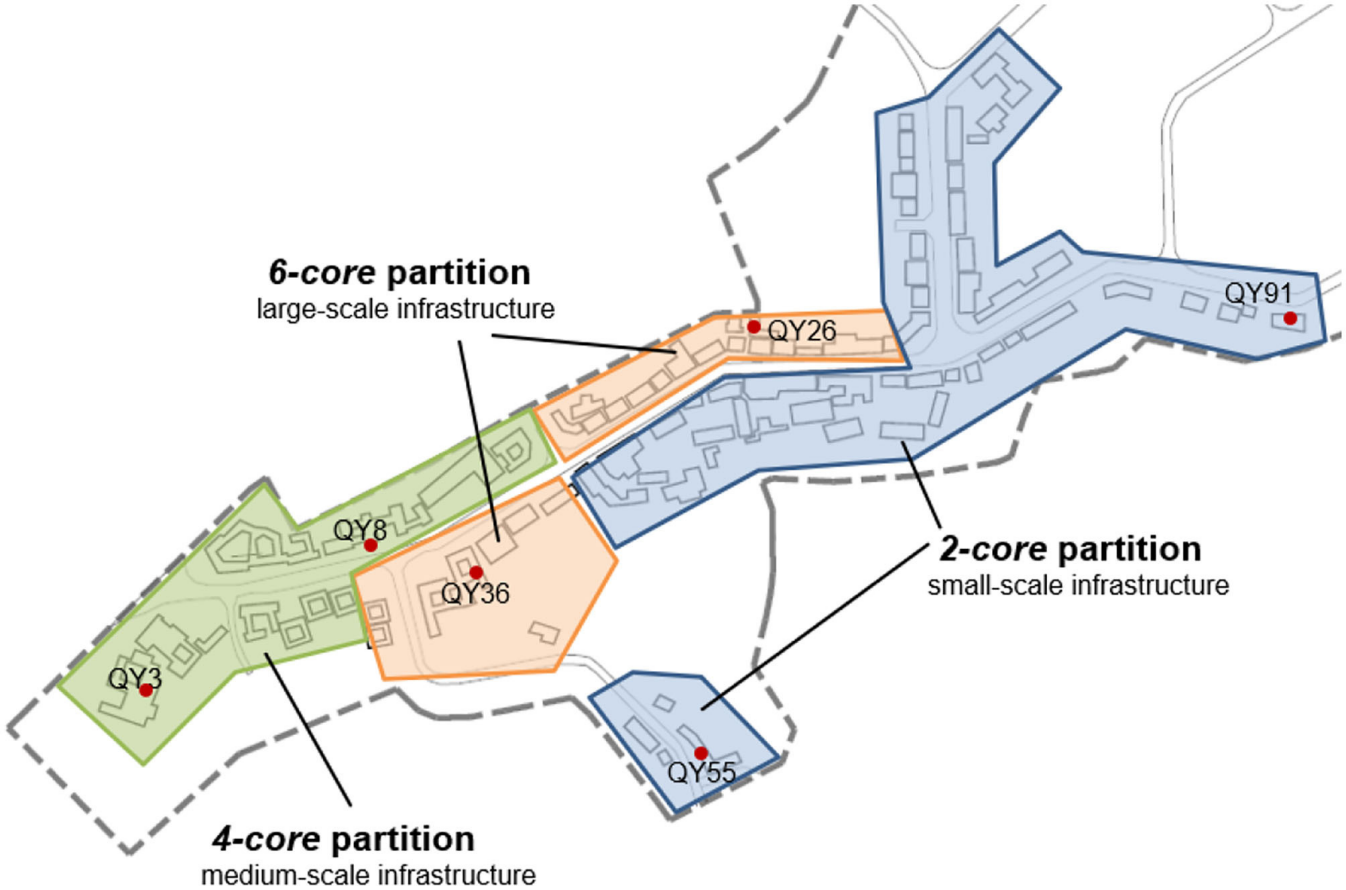
Three-class conservation ranges and corresponding scales of public infrastructure and activity in Qingyang (QY) town based on the analysis of *k-core* indicator.

The increasing spatial connection of historical towns can be realized by design of public facilities and activity places, which are helpful to the SSNS and conservation of the built environment (43, 44). To plan these infrastructures properly, it is necessary to classify the conservation area of the historical town into different conservation grades. In this study, three conservation grades would be classified by the SSNS and environmental elements. Furthermore, the spatial distribution of infrastructures with different scales should be designed by classified conservation grades. For example, as shown in Figure 5, the no <*6-core* households, such as QY26 and QY36 in QY town, can be identified as core conservation nodes for the social network. The space scope of these nodes and historical buildings/sites can be designated as the first-class conservation zone of this town. Large-scale infrastructures, such as shops, schools, and hospitals, can be planned in this zone. Accordingly, main activity spaces as squares, parks, or sport fields can be set. Moreover, the *4-core* households QY3 and QY8 were identified as minor conservation nodes for the social network, and their built environment with historical elements can be classified as the secondary conservation zone of this town. In the zone, medium-scale infrastructures (e.g., retail stores or teahouses) and subordinate activity spaces (e.g., corridors or gardens) could be designed. In addition, the *2-core* households QY91 and QY55 in the historical town are identified as non-key conservation nodes of the social network, and their spatial scope can be identified as the third-class conservation zone of this town. Small-scale infrastructures and public activity spaces, such as small shops, courtyards, and sport equipment areas, could be planned in the zone.

**Figure 6:**
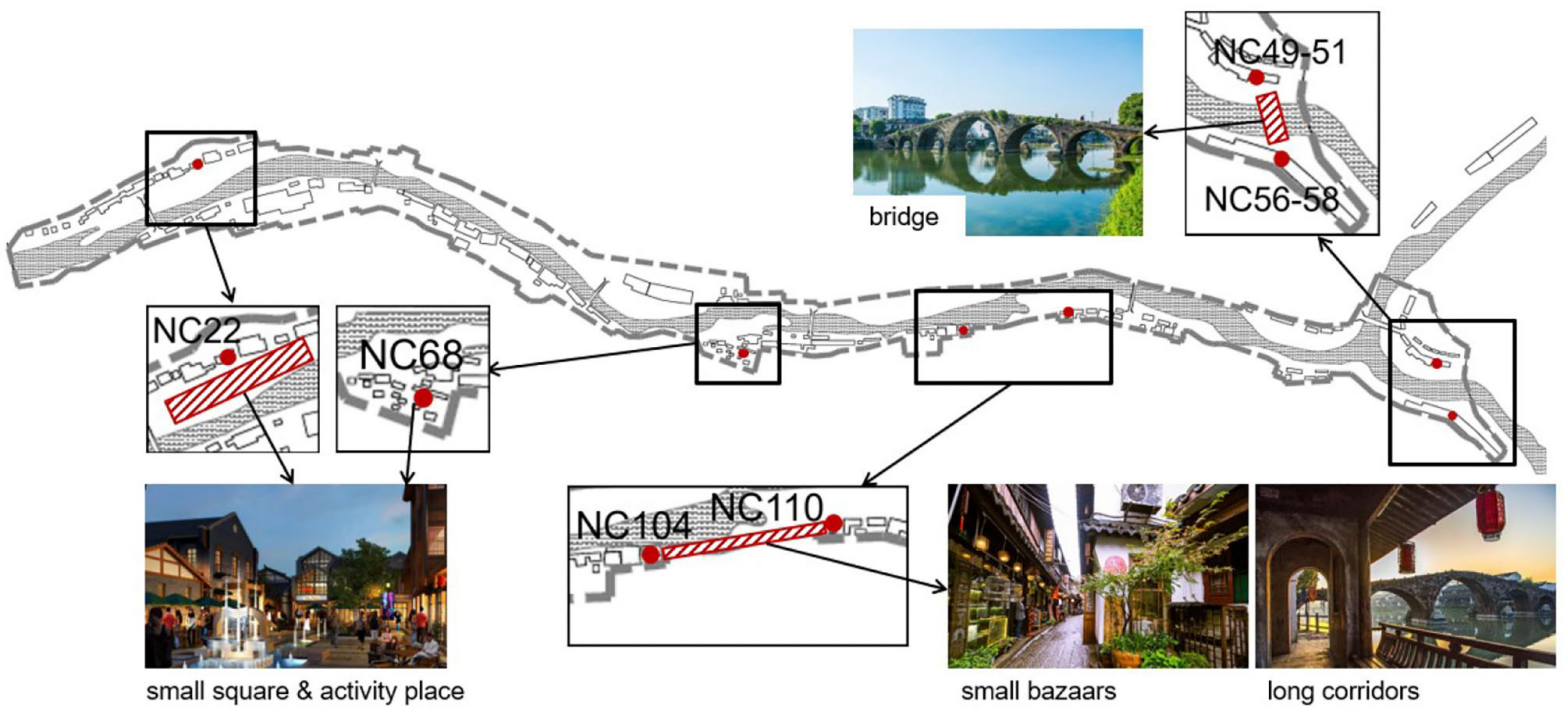
Strategies of the added public infrastructures and activity places in Ningchang town.

In addition, newly-built traffic infrastructures or activity places may help to increase the spatial connection and enhance the SSNS in historical towns (44). Some historical towns with lower SSNS were attributed to the obstruction of natural mountains and rivers on the built environment. Bridges, squares, or bazaars could reduce the barrier of establishment of social ties among households. For example, in NC town (Figure 6), a bridge can be constructed between households NC56 and NC58, and NC49 and NC51. A small square and activity place could be built around households NC22 and NC68. A small bazaar or a long corridor could be added between households NC104 and NC110 or households NC42 and NC38.

### Social Activity Guidance for Social Networks

The social network structure of households in historical towns with higher commercial development and lots of immigration of residents was mainly based on geography or job relationship, such as XT town. It is required to increase the possibility of social ties among residents by increasing the social activities among households so as to improve the SSNS in these historical towns. For new inhabitants who are geographically or professionally related, they can establish interest groups, business associations, or colleague relationships to carry out extensive interest activities, fellowship, league building, and other public activities. It can help like-minded households realize the commonness and dependability in each other, and promote the establishment of social ties (45).

To enhance the stability of social network, adaptive functions should also be carried out to guide social activities of residents in public spaces according to different needs of households in the historical towns (46). For example, some existing households are young people and young couples in BS town. Accordingly, in the conservation planning of this town, it is necessary to strengthen the connection of households with the same property. More public spaces for trendy activities can be placed around household with young people gathering, such as households BS38, BS54, BS66, and BS79. These public spaces will also drive the participation of surrounding households BS85 and BS46 and guide residents to discuss affairs together. At the same time, suitable activity spaces for children can also be placed around households with many young couples, such as households BS46, BS2, BS23, and BS109. Neighbor relationships could be extended to townsman relationships by organizing a block-wide communal event.

Regarding historical towns with high proportion of residential functions, attention should be paid to conservation of large families with kinship structure. In historical towns, families with strong organization and high frequency of crowd interaction can be used to form closed community circles or groups through family activities (47). It can strengthen the existing kinship or geographical relationship and encourage functional mixing or diversified activities to form other social connections.

## Discussion and Conclusions

This article explored the SSNS and the conservation strategy of historical towns based on the construction of social network models and analysis of the *k-core* indicators of seven historical towns in Chongqing, China. The major conclusions can be summarized according to the following four aspects:

(a) Social networks with a multi-cluster or a ring centrality structure (the centripetal structure) had greater stability, while social networks with a series or a fan-shaped dendritic structure (the divergent structure) had lower stability.

Sorting order percentages of the SSNS for the seven historical towns of no <*6-core* is BS town (93.04%) > ZS town (92.68%) > PY town (78.48%) > WQ town (89.9%) > QY town (41.43%) > NC town (36.73%) > XT town (none). It illustrates that the social networks of historical towns with a centripetal-shaped structure (i.e., ZS, BS, WQ, PY, and QY towns) were more stable than historical towns with a divergent-shaped structure (i.e., NC and XT towns).

(b) The SSNS was influenced by spatial layout forms and function of households. The SSNS of historical towns with a group-banded layout and a multi-cluster (centrality) structure was higher than that of towns with other spatial layout forms. The town with residential-dominated function (93.04%), had the highest SSNS (percentage of no <*6-core* was 98.28%).

The spatial layout form of the historical towns might be limited by the terrain of mountains and rivers, which has a great impact on the construction of social ties among households, thus affecting the SSNS. Construction of social ties was influenced by the residential and commercial functions of households, thus affecting the SSNS. To improve the conservation of SSNS in the historical towns, social ties among households need to be increased by enhanced spatial connection and social activities in the built environment and social networks.

(c) Improving the SSNS in the historical towns needs to increase the social ties among households with increasing spatial connection in the built environment and social activities for social networks. The spatial layout of the historical towns can be classified into different conservation grades based on the analysis of k-core indicators. To enhance the stability of the social network, adaptive functions should also be carried out to guide social activities of residents in public spaces according to different needs of households in the historical towns.

(d) Renewal of public facilities and setting of activity spaces could also be planned as classified conservation grades. These could also improve the barrier effect of rivers and mountains on the spatial connection of households. Social activities should be increased to promote the possibility of social ties among households.

The increasing spatial connection of historical towns can be realized by the design of public facilities and activity places, which are helpful to the SSNS and conservation of the built environment. To plan these infrastructures properly, it is necessary to classify the conservation area of a historical town into different conservation grades. In addition, newly-built traffic infrastructures or activity places may help increase spatial connection and enhance the SSNS in the historical towns.

This study focuses on the comparison of SSNS in different historical towns. However, this study also has limitations, particularly regarding small sample size and limited social ties. Under the influence of urbanization, social and economic transformations, and loose interpersonal relationships, social ties in historical towns are also constantly changing. Therefore, it is advisable to compare and analyze changes in social ties of historical towns at different time points in the future, so as to dynamically determine conservation strategies according to the social network conservation of historical towns.

## Data Availability Statement

The original contributions presented in the study are included in the article/supplementary material, further inquiries can be directed to the corresponding authors.

## Author Contributions

All authors listed have made a substantial, direct, and intellectual contribution to the work and approved it for publication.

## Funding

This study was supported by the National Key R&D Program of China (2018YFD1100804).

## Conflict of Interest

The authors declare that the research was conducted in the absence of any commercial or financial relationships that could be construed as a potential conflict of interest.

## Publisher's Note

All claims expressed in this article are solely those of the authors and do not necessarily represent those of their affiliated organizations, or those of the publisher, the editors and the reviewers. Any product that may be evaluated in this article, or claim that may be made by its manufacturer, is not guaranteed or endorsed by the publisher.
